# Dichlorido(6-methyl-2,2′-bipyridine-κ^2^ *N*,*N*′)mercury(II)

**DOI:** 10.1107/S1600536808032777

**Published:** 2008-10-15

**Authors:** Roya Ahmadi, Amin Ebadi, Khadijeh Kalateh, Ali Norouzi, Vahid Amani

**Affiliations:** a Islamic Azad University, Shahr-e-Rey Branch, Tehran, Iran; b Department of Chemistry, Islamic Azad University, Kazeroon Branch, Kazeroon, Fars, Iran; c Islamic Azad University, Izeh Branch, Izeh, Khozestan, Iran

## Abstract

In the mol­ecule of the title compound, [HgCl_2_(C_11_H_10_N_2_)], the Hg^II^ atom is four-coordinated in a distorted tetra­hedral configuration by two N atoms from a 6-methyl-2,2′-bipyridine ligand and two Cl atoms. There is a π–π contact between the pyridine rings [centroid–centroid distance = 3.9758 (5) Å].

## Related literature

For related literature, see: Ahmadi, Kalateh, Ebadi *et al.* (2008 ▶); Ahmadi, Khalighi *et al.* (2008 ▶); Ahmadi, Kalateh, Abedi *et al.* (2008 ▶); Kalateh, Ahmadi *et al.* (2008 ▶); Kalateh, Ebadi *et al.* (2008 ▶); Khalighi *et al.* (2008 ▶); Khavasi *et al.* (2008 ▶); Tadayon Pour *et al.* (2008 ▶); Yousefi, Rashidi Vahid *et al.* (2008 ▶); Yousefi, Tadayon Pour *et al.* (2008 ▶); Yousefi, Khalighi *et al.* (2008 ▶). For related structures, see: Chen *et al.* (2006 ▶); Liu *et al.* (2004 ▶).
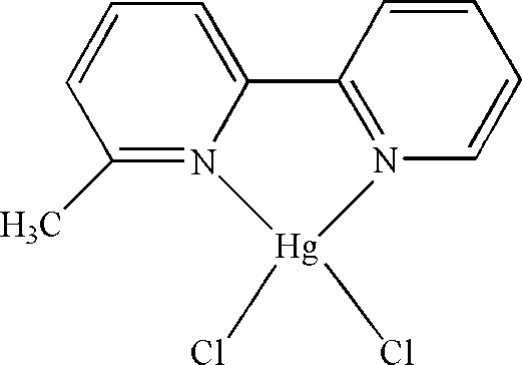


## Experimental

### 

#### Crystal data

[HgCl_2_(C_11_H_10_N_2_)]
*M* *_r_* = 441.70
Monoclinic, 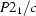

*a* = 9.4742 (19) Å
*b* = 16.164 (3) Å
*c* = 8.2107 (16) Å
β = 105.70 (3)°
*V* = 1210.4 (4) Å^3^
*Z* = 4
Mo *K*α radiation
μ = 13.13 mm^−1^
*T* = 120 (2) K
0.50 × 0.15 × 0.09 mm

#### Data collection

Bruker SMART CCD area-detector diffractometer
Absorption correction: numerical *via* shape of crystal determined optically (**X-SHAPE** and **X-RED**; Stoe & Cie, 2005 ▶) *T*_min_ = 0.108, *T*_max_ = 0.307
14334 measured reflections
3263 independent reflections
2925 reflections with *I* > 2σ(*I*)
*R*_int_ = 0.100

#### Refinement

*R*[*F* ^2^ > 2σ(*F* ^2^)] = 0.092
*wR*(*F* ^2^) = 0.197
*S* = 1.14
3263 reflections
147 parameters
H-atom parameters constrained
Δρ_max_ = 1.39 e Å^−3^
Δρ_min_ = −1.12 e Å^−3^

### 

Data collection: *SMART* (Bruker, 1998 ▶); cell refinement: *SAINT* (Bruker, 1998 ▶); data reduction: *SAINT*; program(s) used to solve structure: *SHELXTL* (Sheldrick, 2008 ▶); program(s) used to refine structure: *SHELXTL*; molecular graphics: *ORTEP-3 for Windows* (Farrugia, 1997 ▶); software used to prepare material for publication: *WinGX* (Farrugia, 1999 ▶).

## Supplementary Material

Crystal structure: contains datablocks I, global. DOI: 10.1107/S1600536808032777/hk2551sup1.cif

Structure factors: contains datablocks I. DOI: 10.1107/S1600536808032777/hk2551Isup2.hkl

Additional supplementary materials:  crystallographic information; 3D view; checkCIF report

## Figures and Tables

**Table:** 

Cl1—Hg1	2.438 (2)
Cl2—Hg1	2.423 (3)
N1—Hg1	2.394 (9)
N2—Hg1	2.297 (10)

**Table:** 

Cl2—Hg1—Cl1	112.32 (10)
N1—Hg1—Cl1	103.4 (2)
N1—Hg1—Cl2	121.1 (2)
N2—Hg1—Cl1	132.8 (2)
N2—Hg1—Cl2	109.8 (2)
N2—Hg1—N1	71.0 (3)

**Fig. 1.**
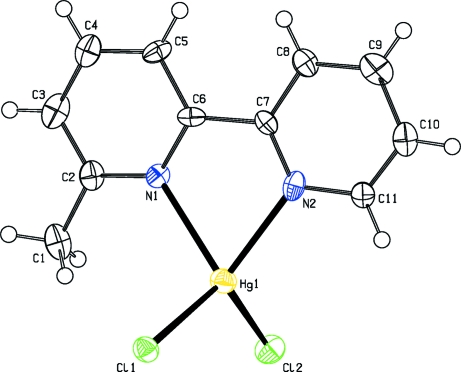
The molecular structure of the title molecule, with the atom-numbering scheme. Displacement ellipsoids are drawn at the 50% probability level.

**Fig. 2.**
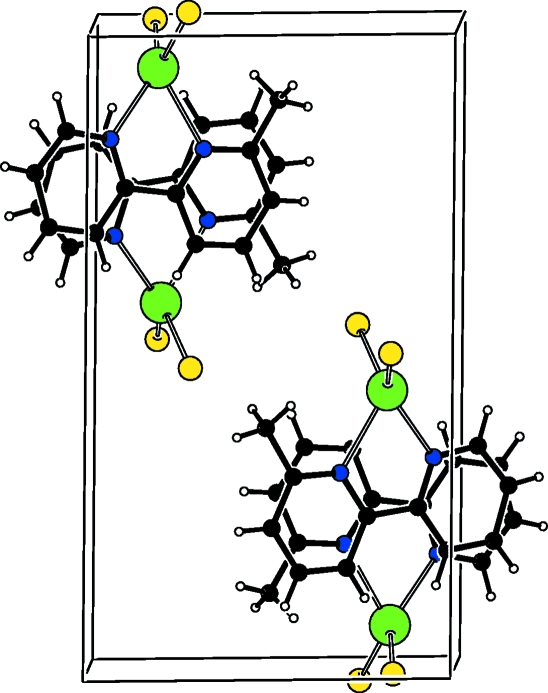
A partial packing diagram of the title compound.

## supplementary crystallographic information

### Comment

Recently, we reported the syntheses and crystal structures of [Zn(5,5'-dmbpy)Cl_2_], (II), (Khalighi *et al.*, 2008), [Zn(6-mbpy)Cl_2_], (III), (Ahmadi, Kalateh, Ebadi *et al.*, 2008), [HgI_2_(4,4'-dmbpy)], (IV), (Yousefi, Tadayon Pour *et al.*, 2008), [Cd(5,5'-dmbpy)(µ-Cl)_2_]_n_, (V), (Ahmadi, Khalighi *et al.*, 2008), [Hg(5,5'-dmbpy)I_2_], (VI), (Tadayon Pour *et al.*, 2008), [Cu(5,5'-dcbpy)(en)(H_2_O)_2_].2.5H_2_O, (VII), (Yousefi, Khalighi *et al.*, 2008), [Hg(dmphen)I_2_], (VIII), (Yousefi, Rashidi Vahid *et al.*, 2008), [In(4,4'-dmbpy)Cl_3_(DMSO)], (IX), (Ahmadi, Kalateh, Abedi *et al.*, 2008), [In(5,5'-dmbpy)Cl_3_(MeOH)], (X), (Kalateh, Ahmadi *et al.*, 2008), {[HgCl(dm4bt)]_2_(µ-Cl)_2_}, (XI), (Khavasi *et al.*, 2008) and {[HgBr(4,4'-dmbpy)]_2_(µ-Br)_2_}, (XII), (Kalateh, Ebadi *et al.*, 2008) [where 5,5'-dmbpy is 5,5'-dimethyl-2,2'-bipyridine, 6-mbpy is 6-methyl-2,2'-bipyridine, 4,4'-dmbpy is 4,4'-dimethyl-2,2' -bipyridine, 5,5'-dcbpy is 2,2'-bipyridine-5,5'-dicarboxylate, en is ethylene- diamine, dmphen is 4,7-diphenyl-1,10-phenanthroline, DMSO is dimethyl sulfoxide and dm4bt is 2,2'-dimethyl-4,4'-bithiazole]. There are several Hg^II^ complexes, with formula, [HgCl_2_(N—N)], such as [HgCl_2_(bipy)], (XIII) and [HgCl_2_(bipy)][HgCl_2_], (XIV), (Chen *et al.*, 2006) and [HgCl_2_(dpdmbip)].CH_2_Cl_2_, (XV), (Liu *et al.*, 2004) [where bipy is 2,2'-bipyridine and dpdmbip is 4,4'-diphenyl-6,6'-dimethyl-2,2'-bipyrimidine] have been synthesized and characterized by single-crystal X-ray diffraction methods. We report herein the synthesis and crystal structure of the title compound, (I).

In the title compound, (I), (Fig. 1), the Hg^II^ atom is four-coordinated in a distorted tetrahedral configuration by two N atoms from 6-methyl-2,2'-bipyridine and two Cl atoms. The Hg—Cl and Hg—N bond lengths and angles (Table 1) are within normal ranges, as in (XIV) and (XV).

In the crystal structure, the π-π contact (Fig. 2) between the pyridine rings, Cg2—Cg3^i^ [symmetry code: (i) x, 1/2 - y, -1/2 + z, where Cg2 and Cg3 are centroids of the rings (N1/C2-C6) and (N2/C7-C11), respectively] may stabilize the structure, with centroid-centroid distance of 3.9758 (5) Å.

### Experimental

For the preparation of the title compound, (I), a solution of 6-methyl-2,2'-bipyridine (0.15 g, 0.88 mmol) in methanol (10 ml) was added to a solution of HgCl_2_ (0.24 g, 0.88 mmol) in acetonitrile (30 ml) and the resulting colorless solution was stirred for 20 min at 313 K. Then, it was left to evaporate slowly at room temperature. After one week, colorless needle crystals of the title compound were isolated (yield; 0.28 g, 72.03%).

### Refinement

H atoms were positioned geometrically, with C-H = 0.93 and 0.96 Å for aromatic and methyl H, respectively, and constrained to ride on their parent atoms with U_iso_(H) = xU_eq_(C), where x = 1.5 for methyl H and x = 1.2 for all other H atoms.

### Crystal data

**Table:**

[HgCl_2_(C_11_H_10_N_2_)]	*F*(000) = 816
*M**_r_* = 441.70	*D*_x_ = 2.424 Mg m^−^^3^
Monoclinic, *P*2_1_/*c*	Mo *K*α radiation, λ = 0.71073 Å
Hall symbol: -P 2ybc	Cell parameters from 1234 reflections
*a* = 9.4742 (19) Å	θ = 2.2–29.2°
*b* = 16.164 (3) Å	µ = 13.13 mm^−^^1^
*c* = 8.2107 (16) Å	*T* = 120 K
β = 105.70 (3)°	Needle, colorless
*V* = 1210.4 (4) Å^3^	0.50 × 0.15 × 0.09 mm
*Z* = 4	

### Data collection

**Table:**

Bruker SMART CCD area-detector diffractometer	3263 independent reflections
Radiation source: fine-focus sealed tube	2925 reflections with *I* > 2σ(*I*)
graphite	*R*_int_ = 0.100
φ and ω scans	θ_max_ = 29.2°, θ_min_ = 2.2°
Absorption correction: numerical *via* shape of crystal determined optically (*X-SHAPE* and *X-RED*; Stoe & Cie, 2005)	*h* = −12→11
*T*_min_ = 0.108, *T*_max_ = 0.307	*k* = −22→22
14334 measured reflections	*l* = −9→11

### Refinement

**Table:**

Refinement on *F*^2^	Secondary atom site location: difference Fourier map
Least-squares matrix: full	Hydrogen site location: inferred from neighbouring sites
*R*[*F*^2^ > 2σ(*F*^2^)] = 0.092	H-atom parameters constrained
*wR*(*F*^2^) = 0.197	*w* = 1/[σ^2^(*F*_o_^2^) + (0.1638*P*)^2^ + 3.9978*P*] where *P* = (*F*_o_^2^ + 2*F*_c_^2^)/3
*S* = 1.14	(Δ/σ)_max_ = 0.005
3263 reflections	Δρ_max_ = 1.39 e Å^−^^3^
147 parameters	Δρ_min_ = −1.12 e Å^−^^3^
0 restraints	Extinction correction: SHELXTL (Sheldrick, 2008), Fc^*^=kFc[1+0.001xFc^2^λ^3^/sin(2θ)]^-1/4^
Primary atom site location: structure-invariant direct methods	Extinction coefficient: 0.020 (2)

### Special details

**Table:**

Geometry. All e.s.d.'s (except the e.s.d. in the dihedral angle between two l.s. planes) are estimated using the full covariance matrix. The cell e.s.d.'s are taken into account individually in the estimation of e.s.d.'s in distances, angles and torsion angles; correlations between e.s.d.'s in cell parameters are only used when they are defined by crystal symmetry. An approximate (isotropic) treatment of cell e.s.d.'s is used for estimating e.s.d.'s involving l.s. planes.
Refinement. Refinement of *F*^2^ against ALL reflections. The weighted *R*-factor *wR* and goodness of fit *S* are based on *F*^2^, conventional *R*-factors *R* are based on *F*, with *F* set to zero for negative *F*^2^. The threshold expression of *F*^2^ > σ(*F*^2^) is used only for calculating *R*-factors(gt) *etc*. and is not relevant to the choice of reflections for refinement. *R*-factors based on *F*^2^ are statistically about twice as large as those based on *F*, and *R*- factors based on ALL data will be even larger.

### Fractional atomic coordinates and isotropic or equivalent isotropic displacement parameters (Å^2^)

**Table:**

	*x*	*y*	*z*	*U*_iso_*/*U*_eq_	
Hg1	0.81647 (4)	0.05913 (2)	0.29276 (5)	0.0248 (3)	
Cl1	0.8330 (3)	−0.00698 (17)	0.5642 (3)	0.0244 (5)	
Cl2	0.7288 (3)	−0.03469 (19)	0.0569 (4)	0.0299 (6)	
N1	0.6912 (9)	0.1861 (5)	0.3103 (11)	0.0187 (16)	
N2	0.9427 (10)	0.1664 (6)	0.2138 (11)	0.0227 (18)	
C1	0.4897 (12)	0.1134 (9)	0.3776 (14)	0.032 (2)	
H1A	0.4493	0.0873	0.2700	0.048*	
H1B	0.4123	0.1261	0.4285	0.048*	
H1C	0.5585	0.0767	0.4500	0.048*	
C2	0.5650 (11)	0.1903 (7)	0.3534 (13)	0.024 (2)	
C3	0.5077 (11)	0.2686 (8)	0.3770 (14)	0.030 (2)	
H3	0.4196	0.2725	0.4058	0.036*	
C4	0.5820 (12)	0.3385 (8)	0.3573 (14)	0.029 (2)	
H4	0.5457	0.3900	0.3764	0.034*	
C5	0.7106 (11)	0.3335 (6)	0.3093 (15)	0.024 (2)	
H5	0.7601	0.3811	0.2924	0.028*	
C6	0.7649 (11)	0.2543 (6)	0.2868 (11)	0.0175 (17)	
C7	0.9018 (10)	0.2430 (6)	0.2327 (11)	0.0175 (17)	
C8	0.9819 (11)	0.3114 (7)	0.2004 (15)	0.025 (2)	
H8	0.9522	0.3648	0.2181	0.030*	
C9	1.1063 (13)	0.2995 (8)	0.1417 (14)	0.031 (2)	
H9	1.1609	0.3437	0.1191	0.037*	
C10	1.1450 (11)	0.2146 (8)	0.1183 (13)	0.028 (2)	
H10	1.2258	0.2029	0.0784	0.033*	
C11	1.0615 (10)	0.1513 (7)	0.1553 (12)	0.0211 (19)	
H11	1.0870	0.0969	0.1399	0.025*	

### Atomic displacement parameters (Å^2^)

**Table:**

	*U*^11^	*U*^22^	*U*^33^	*U*^12^	*U*^13^	*U*^23^
Hg1	0.0291 (4)	0.0223 (4)	0.0234 (4)	−0.00241 (12)	0.0075 (2)	−0.00050 (12)
Cl1	0.0251 (11)	0.0254 (12)	0.0226 (10)	0.0004 (9)	0.0061 (8)	0.0033 (9)
Cl2	0.0307 (13)	0.0297 (13)	0.0264 (12)	−0.0031 (11)	0.0032 (10)	−0.0077 (11)
N1	0.012 (3)	0.021 (4)	0.020 (4)	−0.001 (3)	0.000 (3)	−0.003 (3)
N2	0.022 (4)	0.032 (5)	0.014 (3)	0.001 (3)	0.004 (3)	−0.007 (3)
C1	0.019 (4)	0.050 (7)	0.023 (5)	−0.005 (5)	0.002 (4)	0.002 (5)
C2	0.014 (4)	0.034 (5)	0.023 (5)	0.002 (4)	0.001 (3)	0.002 (4)
C3	0.015 (4)	0.044 (7)	0.026 (5)	0.001 (4)	−0.003 (4)	−0.012 (5)
C4	0.025 (5)	0.039 (6)	0.020 (4)	0.005 (4)	0.003 (4)	−0.004 (4)
C5	0.018 (4)	0.018 (4)	0.031 (5)	0.003 (3)	0.000 (4)	−0.005 (4)
C6	0.025 (4)	0.014 (4)	0.008 (3)	0.000 (3)	−0.005 (3)	0.001 (3)
C7	0.018 (4)	0.023 (4)	0.009 (3)	−0.002 (3)	0.000 (3)	0.004 (3)
C8	0.018 (4)	0.030 (5)	0.025 (5)	−0.006 (4)	0.003 (3)	0.000 (4)
C9	0.025 (5)	0.042 (7)	0.018 (4)	0.004 (4)	−0.008 (4)	0.017 (5)
C10	0.017 (4)	0.045 (7)	0.019 (4)	0.004 (4)	0.001 (3)	0.004 (5)
C11	0.019 (4)	0.027 (5)	0.017 (4)	0.008 (4)	0.003 (3)	0.009 (4)

### Geometric parameters (Å, °)

**Table:**

Cl1—Hg1	2.438 (2)	C5—C6	1.410 (13)
Cl2—Hg1	2.423 (3)	C5—H5	0.9300
N1—Hg1	2.394 (9)	C6—N1	1.347 (13)
N2—Hg1	2.297 (10)	C6—C7	1.492 (14)
C1—C2	1.473 (17)	C7—N2	1.319 (14)
C1—H1A	0.9600	C7—C8	1.406 (14)
C1—H1B	0.9600	C8—C9	1.402 (17)
C1—H1C	0.9600	C8—H8	0.9300
C2—N1	1.338 (13)	C9—C10	1.446 (18)
C2—C3	1.411 (17)	C9—H9	0.9300
C3—C4	1.363 (18)	C10—C11	1.377 (16)
C3—H3	0.9300	C10—H10	0.9300
C4—C5	1.381 (16)	C11—N2	1.360 (13)
C4—H4	0.9300	C11—H11	0.9300
			
Cl2—Hg1—Cl1	112.32 (10)	C3—C4—C5	120.6 (11)
N1—Hg1—Cl1	103.4 (2)	C3—C4—H4	119.7
N1—Hg1—Cl2	121.1 (2)	C5—C4—H4	119.7
N2—Hg1—Cl1	132.8 (2)	C4—C5—C6	118.1 (10)
N2—Hg1—Cl2	109.8 (2)	C4—C5—H5	121.0
N2—Hg1—N1	71.0 (3)	C6—C5—H5	121.0
C2—N1—Hg1	123.6 (7)	N1—C6—C5	120.3 (10)
C2—N1—C6	122.1 (9)	N1—C6—C7	117.9 (9)
C6—N1—Hg1	114.1 (6)	C5—C6—C7	121.8 (9)
C7—N2—Hg1	118.9 (7)	N2—C7—C8	121.7 (9)
C7—N2—C11	120.4 (10)	N2—C7—C6	117.2 (9)
C11—N2—Hg1	120.6 (8)	C8—C7—C6	121.1 (9)
C2—C1—H1A	109.5	C9—C8—C7	120.2 (11)
C2—C1—H1B	109.5	C9—C8—H8	119.9
H1A—C1—H1B	109.5	C7—C8—H8	119.9
C2—C1—H1C	109.5	C8—C9—C10	116.3 (11)
H1A—C1—H1C	109.5	C8—C9—H9	121.8
H1B—C1—H1C	109.5	C10—C9—H9	121.8
N1—C2—C3	119.1 (10)	C11—C10—C9	119.6 (10)
N1—C2—C1	119.5 (10)	C11—C10—H10	120.2
C3—C2—C1	121.3 (10)	C9—C10—H10	120.2
C4—C3—C2	119.8 (11)	N2—C11—C10	121.7 (10)
C4—C3—H3	120.1	N2—C11—H11	119.2
C2—C3—H3	120.1	C10—C11—H11	119.2
			
C2—N1—Hg1—Cl1	−51.3 (8)	C5—C6—C7—C8	−0.5 (13)
C2—N1—Hg1—Cl2	75.5 (8)	N2—C7—C8—C9	2.0 (15)
C2—N1—Hg1—N2	177.6 (8)	C6—C7—C8—C9	−176.9 (9)
C6—N1—Hg1—Cl1	123.3 (6)	C7—C8—C9—C10	−0.2 (14)
C6—N1—Hg1—Cl2	−109.9 (6)	C8—C9—C10—C11	−0.7 (14)
C6—N1—Hg1—N2	−7.7 (6)	C9—C10—C11—N2	0.0 (15)
C7—N2—Hg1—Cl1	−83.2 (8)	C3—C2—N1—C6	−0.7 (14)
C7—N2—Hg1—Cl2	124.8 (7)	C1—C2—N1—C6	−179.9 (9)
C7—N2—Hg1—N1	7.6 (7)	C3—C2—N1—Hg1	173.6 (7)
C11—N2—Hg1—Cl1	94.0 (7)	C1—C2—N1—Hg1	−5.7 (13)
C11—N2—Hg1—Cl2	−58.0 (7)	C5—C6—N1—C2	0.7 (14)
C11—N2—Hg1—N1	−175.2 (8)	C7—C6—N1—C2	−177.8 (8)
N1—C2—C3—C4	−0.7 (16)	C5—C6—N1—Hg1	−174.0 (7)
C1—C2—C3—C4	178.5 (10)	C7—C6—N1—Hg1	7.5 (10)
C2—C3—C4—C5	2.0 (16)	C8—C7—N2—C11	−2.7 (14)
C3—C4—C5—C6	−2.0 (16)	C6—C7—N2—C11	176.2 (8)
C4—C5—C6—N1	0.6 (14)	C8—C7—N2—Hg1	174.5 (7)
C4—C5—C6—C7	179.1 (9)	C6—C7—N2—Hg1	−6.6 (11)
N1—C6—C7—N2	−1.0 (12)	C10—C11—N2—C7	1.7 (14)
C5—C6—C7—N2	−179.4 (9)	C10—C11—N2—Hg1	−175.4 (7)
N1—C6—C7—C8	177.9 (9)		
